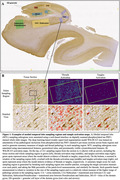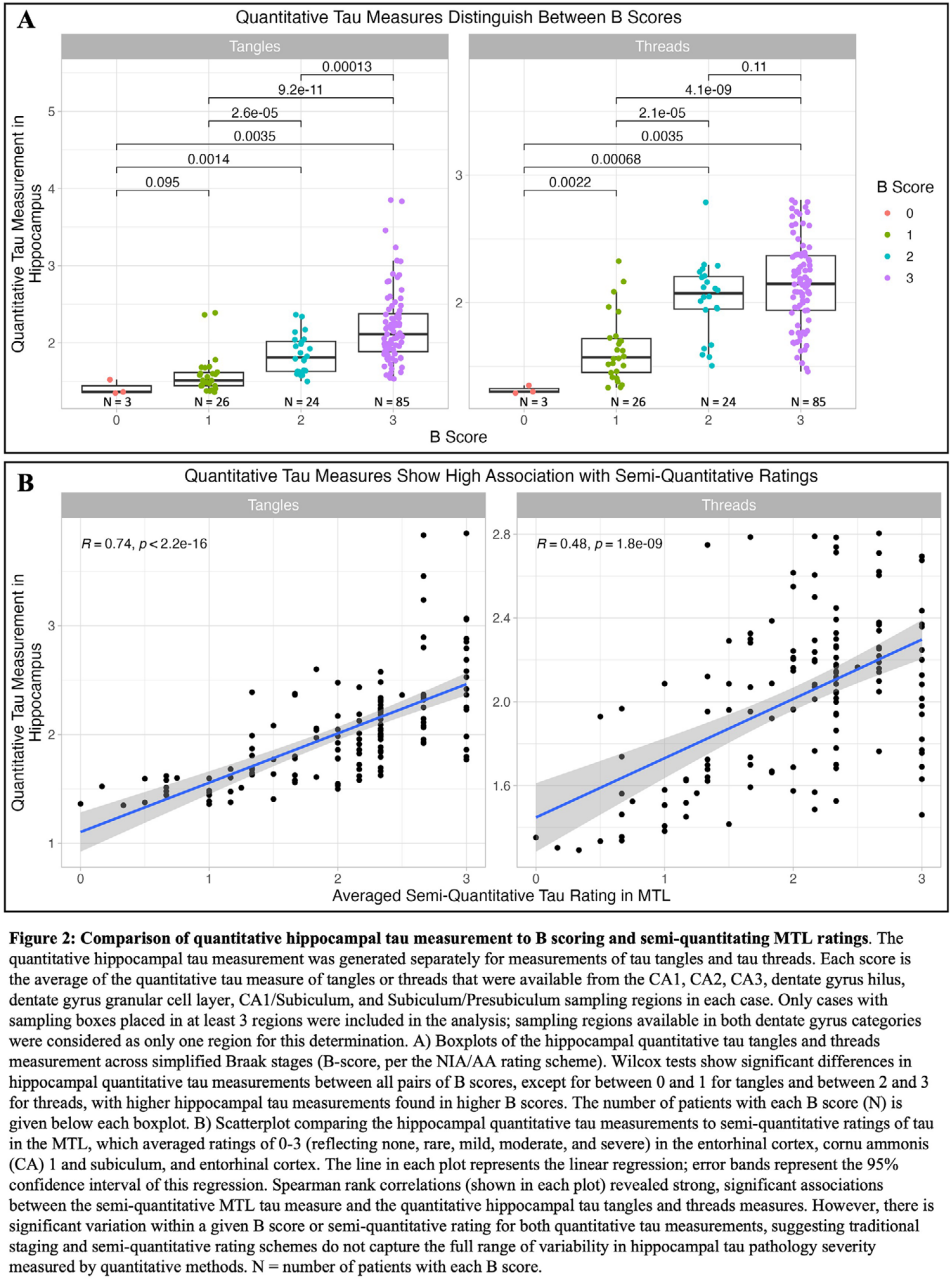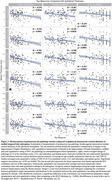# Association of quantitative measurements of postmortem tau tangle and thread pathology with antemortem cortical thickness measurements in neuropathologically confirmed Alzheimer’s Disease continuum patients

**DOI:** 10.1002/alz.093722

**Published:** 2025-01-09

**Authors:** Amanda E Denning, Ranjit Ittyerah, Niyousha Sadeghpour, Lisa M Levorse, Eunice Chung, Chinmayee Athalye, Sadhana Ravikumar, Mengjin Dong, Michael Tran Duong, Yue Li, Ademola Ilesanmi, Stanislau Hrybouski, Lasya P Sreepada, Philip Sabatini, MaKayla Lowe, Alejandra Bahena, Ryohei Watanabe, Boram Kim, Robin de Flores, John L. Robinson, Theresa Schuck, Daniel T Ohm, Sanaz Arezoumandan, Ricardo Insausti, Laura E.M. Wisse, David J Irwin, Eddie B Lee, Sandhitsu R. Das, David A Wolk, Paul A. Yushkevich

**Affiliations:** ^1^ University of Pennsylvania, Philadelphia, PA USA; ^2^ Normandie Univ, UNICAEN, INSERM, U1237, PhIND Physiopathology and Imaging of Neurological Disorders, NeuroPresage Team, GIP Cyceron, Caen France; ^3^ University of Castilla‐La Mancha, Albacete Spain; ^4^ Department of Clinical Sciences Lund, Lund University, Lund, Lund Sweden

## Abstract

**Background:**

Tau pathology and neurodegeneration in the medial temporal lobe (MTL) are highly associated in Alzheimer’s Disease (AD). However, the spatial pattern of neurodegeneration, contribution of individual tau inclusion types, and influence of MTL co‐pathologies (i.e., TDP‐43) remain poorly understood. Traditional semi‐quantitative ratings or staging schemes of tau pathology capture limited variability in severity and provide no differentiation between inclusion types (i.e., tangles, threads). We correlate semi‐quantitative and quantitative measures of MTL tau pathology from postmortem tissue samples with MTL cortical thickness measures from antemortem MRI.

**Method:**

Hippocampus histology slides (65.2% male, age 74.09±10.74 years) were available from 138 patients with AD continuum neuropathological diagnoses and antemortem T1‐weighted imaging within 10 years of death. Ipsilateral median cortical thickness measurements in anterior/posterior hippocampus, entorhinal cortex, Brodmann areas 35/36 (BA35/36), and parahippocampal cortex were automatically derived from MRI. We digitally annotated 7 MTL sampling regions on phosphorylated tau PHF1‐stained 6 µm tissue sections and trained a machine learning method to generate summary measures of tau tangle and thread pathology in each region (Fig.1). Quantitative measures of hippocampal tau tangles and threads were compared to simplified Braak staging (B score) and semi‐quantitative neuropathologist ratings of MTL tau. We performed one‐sided Spearman correlations between tau pathology measures and ipsilateral cortical thickness, covaried for age at death, antemortem interval, sex, and semi‐quantitative MTL TDP‐43 rating.

**Result:**

Both quantitative hippocampal tau measures showed significant differences across B score levels and high correlation with semi‐quantitative ratings of MTL tau, but also great variability within each score/rating (Fig.2). Additionally, hippocampal tau tangle and thread pathology measures both showed significant negative correlations with ipsilateral cortical thickness in all MTL subregions, except for the threads measurement with BA35 thickness. Semi‐quantitative tau measures showed negative correlations with thickness in all subregions except BA35. Compared to semi‐quantitative ratings, the associations with quantitative tau measures were generally stronger and statistically more robust (significant difference for tangles/BA35) (Fig.3).

**Conclusion:**

In a large antemortem‐postmortem dataset, quantitative measures of postmortem tau tangle and thread pathology each showed strong, significant association with ipsilateral antemortem cortical thickness across MTL subregions, independent of TDP‐43 pathology and stronger than the association with semi‐quantitative ratings.